# A Review of Australian Animal Welfare Legislation, Regulation, Codes of Practice, and Policy, and Their Influence on Stakeholders Caring for Wildlife and the Animals for Whom They Care

**DOI:** 10.3390/ani9060335

**Published:** 2019-06-09

**Authors:** Bruce Englefield, Simone A. Blackman, Melissa Starling, Paul D. McGreevy

**Affiliations:** 1 School of Veterinary Science, The University of Sydney, Sydney, NSW 2006, Australia; melissa.starling@sydney.edu.au (M.S.); paul.mcgreevy@sydney.edu.au (P.D.M.); 2 Tasmanian School of Business and Economics and the Faculty of Law, University of Tasmania, Hobart, Tasmania 7005, Australia; Simone.bingham@utas.edu.au

**Keywords:** wildlife, native animals, wildlife care, legislation, mental well-being, physical well-being

## Abstract

**Simple Summary:**

The Australian constitution does not mention native animals. Responsibility for animal welfare is largely retained by the states and territories via a fragmented, complex, contradictory, inconsistent system of regulatory management. The problem this creates for volunteers undertaking the rescue and rehabilitation of native animals is complex. Capturing and rehabilitating wild animals goes against regulations. In most jurisdictions, it is illegal to microchip, band, or mark an animal, making it almost impossible to monitor their survival. A minimum of 50,000 rehabilitated native animals are released back to the wild each year, with few checks afterwards to see how well or if they are surviving. Whilst it can be appropriate to rehabilitate and release injured native animals back to the wild, there may be moral, ethical, and practical reasons for not releasing hand-reared orphan native animals. With no reliable method of identification, no instructions on how to get animals ready for release or see if they are suitable, and little post-release checking, the practice of placing hand-reared native animals into the wild, and the regulatory framework enabling it, should be reviewed.

**Abstract:**

The Australian constitution makes no mention of native animals. Responsibility for animal welfare is largely retained by the states and territories via a fragmented, complex, contradictory, inconsistent system of regulatory management. Given that most jurisdictions have expressly made the possession of wildlife unlawful, the action of taking and possessing an animal, to rehabilitate it, defies the regulatory process. In most jurisdictions, it is illegal to microchip, band, or mark an animal, meaning that no reliable method is available to monitor an animal. Each year, a minimum of 50,000 rehabilitated native animals are released back to the wild, with little post-release monitoring. Where required, the assessments of behavioural and health requirements to confirm suitability for release may be undertaken by people with either negligible or questionable qualifications. Whilst it can be appropriate to rehabilitate and release injured native animals back to the wild, there may be moral, ethical, and practical reasons for not releasing hand-reared orphan native animals. This article examines the evolution, and explains the consequences, of decentralised regulation on wildlife carers and rehabilitating animals. It recommends that the practice of placing hand-reared native animals into the wild, and the regulatory framework that provides for it, should be reviewed.

## 1. Introduction

Australian animal law is similar to the legislation of other nations in that, on the one hand, there are laws that allow extermination, culling, and harming animals while, on the other hand, there are those that protect animals from being killed, harvested, or harmed. A plethora of legislation, regulation, codes of practice, and guidelines are intended to guide the volunteer wildlife carers who rescue and rehabilitate injured and orphaned native wildlife (Table 1). These carers must navigate laws and policies that differ in complexity, aim, and implementation, both between and within Australian states and territories. The well-being of rescued wildlife is a central objective for wildlife carers, but navigating these laws successfully is an additional challenge to that of rehabilitating the injured and orphaned animals.

Native animals play a critical role in the Australian environment and Australia’s identity. A kangaroo (*Macropus rufus*) and emu (*Dromaius novaehollandiae*) feature on the Australian coat-of-arms, while various state and territory coats-of-arms also bear images of kangaroos, in the company of black swans (*Cygnus atratus*), brolgas (*Grus rubicunda*), shrikes (*Laniidae*), wedge-tailed eagles (*Aquila audax*), and Tasmanian tigers (*Thylacinus cynocephalus*). However, the appearance of animals in Australian heraldry emblems seems to have offered no guarantee of the welfare of featured species being promoted by legislators. Indeed, two Tasmanian tigers feature on the Tasmanian (TAS) coat-of-arms, which was granted a royal warrant from King George V, and the current Tasmanian Government logo, issued under the *Trademark Act 1995* (Commonwealth) [40], features a Tasmanian tiger peering through long grass. This logo is now used as an identifying device and a visual communication of the Tasmanian brand [41]. Paradoxically, it was legislation passed by the Tasmanian parliament in 1888, placing a £1 bounty on Tasmanian tigers, that contributed to their extinction. In contrast, some introduced species appear in the heraldry of Queensland (QLD). The QLD Government introduced legislation in 1997 confirming the appearance of the red deer (*Cervus elaphus*) as a feature on the state’s coat-of-arms, despite this being an introduced species. Some 11 years later, the same legislative body declared the red deer as, “invasive animals that are listed as restricted matter”, permitting them to be shot in environmentally sensitive areas [42]. Although others [43,44,45,46] have deliberated about what constitutes an introduced or native species, and some cite the dingo as a blurring distinction [47,48], the dingo is recognised as a native animal under the laws of all Australian jurisdictions. These recognise a native species as one that was present in Australia before the year 1400 [49] (Section 528). By way of contrast, red deer did not exist in Australia until early white settlers introduced them from Europe in the 19th century, confirming them as a non- native species. 

These two instances show that the states use legislation both to identify themselves by aligning with species-specific emblems and also, when it suits, to euthanase members of the very same species. 

Australian states are sovereign powers. The states formed the Commonwealth and delegated to it specific responsibilities, such as defence, income tax, and external treaties. There are no national laws mandating minimum animal welfare requirements. The responsibility for the management of wildlife and other animals remains with the states and territories. However, by way of an exception, there are legally binding national codes of practice for animal welfare in research, i.e., the Australian code for the care and use of animals for scientific purposes [50]. The Commonwealth’s role is limited in this domain because the Australian constitution mentions neither animal welfare nor animals, other than fisheries, in any detail [51].

The Australian philosopher Peter Singer gave direction to the treatment of animals in 1989, with his book *Animal Liberation* [52]. Animal law is an independent and unique discipline and, over the last 10–20 years, has emerged as a focal domain among Australian legal scholars, reflecting societal interest in animal rights, welfare, and protection. Increasingly, law journals are publishing articles on animal law, and several textbooks dedicated to the topic have been written by Australian academics [53,54,55,56,57]. In parallel with this, community expectations and public awareness of animal welfare issues have been heightened by the use of social media and the broadcast of covert video recordings by animal protection organisations in Australia. These trends are likely to continue to grow, despite the so-called ag-gag laws that have been proposed to shut down covert surveillance activities [58]. 

World Animal Protection is an international non-profit animal welfare organization that recently published a classification of 50 countries around the world, according to their commitments to protect animals and improve animal welfare in policy and legislation [59]. Largely due to its lack of a national approach to animal welfare, Australia rated overall as a ‘C’, in contrast to New Zealand, who was rated a superlative ‘A’ on a scale from ‘A’ to ‘G’ (with ‘A’ representing the highest scoring and ‘G’ the most room for improvement). Australia also scored poorly due to its failure to either support the Universal Declaration on Animal Welfare or to legislate Office International des Epizooties (OIE, the World Organisation for Animal Health) welfare standards, as well as perceived deficits in OIE participation and a dearth of education programs. 

Despite Australia’s current failures on the animal welfare front, it is worth noting that a cohesive national approach to animal welfare policy has been attempted in the recent past. The Australian Animal Welfare Strategy (AAWS) was developed to outline directions for future improvements in the welfare of animals and to provide national and international communities with an appreciation of animal welfare arrangements in Australia. The Primary Industries Ministerial Council, a part of the Council of Australian Governments process and the appropriate forum for attention to achieve a national approach and harmonisation of laws, endorsed the AAWS in May 2004 and the first National Implementation Plan for the strategy in May 2006 [60]. From 2010 to 2013, the Commonwealth contributed funding to the AAWS. The AAWS aimed to help build partnerships, improve coordination, reduce duplication of effort, and deliver a more effective and consistent national approach to improving the welfare of animals [61]. However, as part of its 2014–2015 Budget measures, the incoming Liberal–National Party coalition ceased funding of the AAWS. 

An issue that might benefit from a national approach is the injuring and killing of native animals on Australian roads by wildlife vehicle collisions (WVCs), colloquially called roadkill. The annual Australian mammalian roadkill has been conservatively estimated at 4,000,000 animals [62], Subsequent to collisions with vehicles, the numbers of animals that suffer a slow death in the bush is uncountable. WVCs and other events, both anthropogenic and natural, produce a massive burden of injured and orphaned animals, the care of which is often left to volunteers. Voluntary wildlife carers must be competent in understanding the federal and state legislation, regulations, codes of practice, general guides, and guidelines so that they operate within the law (Table 1). Non-compliance has consequences that can prove financially costly as well as emotionally draining. Similarly, training is required so that carers can manage the complex tasks involved in rehabilitating wildlife and continue their professional development with ongoing education.

The noun ‘rehabilitation’ comes from the Latin prefix ‘re’, meaning again and the verb ‘habitare’, meaning to live, hence wildlife rehabilitation is generally defined as: “providing professional care to sick, injured, and orphaned wild animals so ultimately they can be returned to their natural habitat or state” [11] (p. 4 Objectives), [63,64] However, other definitions, both in legislation and in literature change the words “natural habitat or state”, to be more specifically, “the wild”. For example, Tasmanian legislation mentions, “with the goal of releasing them back to the wild” [28] (p. 3 Definitions, bullet point 2), and Australian and USA literature mentions, “to appropriate habitats in the wild” [65,66]. The semantics of using the terms “natural state or habitat” and “the wild” in rehabilitation reveal much more than a pedantic difference. In changing “natural habitat or state” to become “the wild”, laws that mandate the wild for the release of hand-reared animals leave no flexibility to accommodate those animals whose well-being might be optimised by remaining in captivity. It is suggested that a difference should be acknowledged between rescue animals that are injured (having been raised in the wild as their natural habitat or state) and those that are hand-reared (for whom captivity has become their natural habitat or state). 

A history of the establishment of regulation in Australia is presented to explain how the complex regulatory system evolved, how its implementation has produced practices that may be questionable in their efficacy in maximising the well-being of rescued native animals and the people who care for them, and how seemingly minor changes of nomenclature in laws can have far-reaching consequences. Previous reports have considered the complexities of wildlife care in Australia [67,68,69,70,71], but none have considered human and animal welfare in the light of the disjointed legal frameworks that apply across the states and territories. This article provides a review and critique of the current systems of regulating the rescue and management of injured and orphaned native animals in Australia. It explains the consequences of the current decentralised system of regulation for wildlife carers and the animals for which they care. A consideration of regulation from both the USA and the UK is included to either illustrate perceived deficiencies in approaches that exist in Australia or to explore alternatives that might improve the current system. Sections of this review investigate the regulatory processes of:

(a) Rescue and taking of injured and orphaned wildlife;
(b) Licensing, permitting, and training requirements for wildlife carers to keep wildlife;
(c) Facility requirements, standards of care, monitoring and identifying of animals;
(d) Funding and monitoring of carers/carer networks;
(e) Releasing animals;
(f) Pre-release methodology and post-release monitoring.

## 2. Discussion

### 2.1. Rescue and Taking of Injured and Orphaned Wildlife

A system has evolved in Australia involving government, not-for-profit organisations, and volunteer groups managing the rescue and rehabilitation of injured and orphaned animals. Generally, vehicular casualties are reported to local authorities, for example, police, National Parks and Wildlife Service, state infrastructure, and environment departments or to designated organisations via wildlife rescue helplines, for example, Royal Society for the Prevention of Cruelty to Animals Australia (RSPCAA), Wildlife Information, Rescue and Education Service (WIRES), and Wildcare Australia Inc. Information about the casualties is then passed to volunteer wildlife carers and rehabilitators, who take on the animals. 

Regulations relevant to wildlife vehicle collisions (WVCs) vary from a legal requirement to stop and assist and to report the incident in the Australian Capital Territory (ACT) and Victoria (VIC), to no requirement to stop or report in TAS and Western Australia (WA), where any action depends on the morality of the individual driver. In the ACT, for example, it is an offence to kill a native animal and a person commits an offence if they “engage in conduct” that causes the death of a native animal. One might consider that “engaging in conduct” could include driving a vehicle. However, a subsequent clause states that the above does not apply if the death of the animal is caused by an accidental collision with a motor vehicle [5] (Chapter 6, Part 6.1. Division a 6.1.2, 130 {1}]). 

Legal provisions also require the alleviation of an animal’s suffering to be an obligation under the ACT Animal Welfare Act 1992 [1], “a person commits an offence if they injure a live member of a vertebrate species and do not take reasonable steps to alleviate any pain suffered by the animal”. Furthermore, if there is no person in charge of the animal, the person in attendance must report the injury within 72 h. Examples given of animals that may have no person in charge include kangaroos (*Macropus spp*), foxes (*Vulpes vulpes*), and galahs (*Eolophus roseicapilla*), which may effectively be seen as meaning all wildlife. Thus, a vehicle driver who accidentally kills such an animal is exempt but is required to assist and report if the animal is injured. Under legislation proposed in the ACT [72], the requirement to report an accident will change to a requirement to tell a relevant person within 2 h where the animal was injured. An example is given of “circumstances, if a car hits a mammal—the size of the animal, witness accounts that the driver swerved or braked to avoid the animal or stopped after hitting the animal”. 

In all states except WA, it is illegal to take or possess wildlife, dead or alive, whole or parts of, unless exempted by reason of having a permit to do so (Table 2). This disbars the general public from doing any more than rendering assistance to an injured WVC victim. Clearly, they must not remove the animal or its offspring. With the exception of WA, all states and territories require licensing of people wishing to possess wildlife for the purpose of rehabilitation. In TAS, no permit or license is required for taking and keeping Bennett’s wallabies (*Macropus rufogriseus*), pademelons (*Thylogale*), and common brushtail possums (*Trichosurus vulpecula*) (Table 2). In the Northern Territory (NT), ACT, and South Australia (SA) it is also possible to obtain licences to keep, sell, import, and export certain native wildlife on a permanent basis, and in the NT, rescued wildlife becomes the property of the licence owner. In QLD, an animal taken in for rehabilitation under licence ceases to be the property of the state, until released (Table 2). Given that most states have expressly made the possession of wildlife unlawful, the action of taking and possessing an animal, to rehabilitate it, defies the regulatory categories. However, if statutorily provided, a permit or license serves as an exemption. This allows governments to regulate wildlife care and rehabilitation via a licensing system and thus ensure that those who seek to become wildlife carers are adequately qualified, have the necessary finance and facilities to do so, and can operate in a lawful manner. 

### 2.2. Licensing, Permitting, and Training Requirements for Wildlife Carers to Keep Wildlife

Australia started to regulate the activity of wildlife rehabilitation in the 1990s on a state or territory basis, but no national guidance is yet available. All states and territories, except WA, mandate that to undertake the care and rehabilitation of protected wildlife, a licence or permit is required. Protected wildlife is generally defined as native birds, reptiles, amphibians, and mammals, with some exceptions, such as dingoes in NSW [10] and common brushtail possums, Bennett’s wallabies, and Tasmanian pademelons in TAS [27]. NSW legislation attempts to ensure that no rescued wildlife suffers at the hands of untrained personnel by making it compulsory to undertake professional training and comply with the standards in the code of practice. This is a condition for obtaining a license, issued under Section 120 of the National Parks and Wildlife Act 1974. NSW, to practice as a wildlife carer [77]. More advanced training is required within three years, if a licence is to be renewed. Most other states and territories have less structured training requirements, some stating that proof of experience in wildlife care in a permit application is sufficient. The worst-case scenario is in TAS, where any untrained person can take in a rescued common brushtail possum, Bennett’s wallaby, or pademelon, without the requirement for a licence or notification to authorities. 

Licences or permits may be issued to an individual or to an organisation, such as a wildlife shelter or a wildlife care group. By regulating the issuing of a licence or permit, authorities can require and monitor a minimum level of professionalism and preparedness of candidate carers. This level of skill and knowledge can serve as a benchmark in deciding if individuals are ready to take on the complex task of caring for rescued wildlife. 

The Federal/State system of government in the USA is similar to that in Australia, with the regulation of wildlife rehabilitation and care being delegated to individual states. However, in 1982, it was found desirable and possible to create the American National Wildlife Rehabilitators Association (NWRA). This addressed the need to gather and disseminate information on the care of rescued animals and to establish universal national standards. This association is now funded mainly by tax deductible donations and also by some government grants [78]. In the United Kingdom (UK), the centralised system of government facilitates a similarly unified approach through the British Wildlife Rehabilitation Council, which was formed in 1987 and operates as a charitable organisation. Australia still has no equivalent organisation or council, even though its six states and two territories must be more manageable than 50 USA states and 100 UK counties. An Australian Wildlife Rehabilitation Conference has been a biennial event since 2008 but receives no government funding.

In the USA and the UK, having nationally recognised organisations has led to government recognition of the contribution and value of wildlife carers. These organisations have moved to ensure that a minimum standard of preparedness, knowledge, and standard of care is provided to wildlife undergoing rehabilitation. The regulations produced not only provide protection for the animals in care but also for the people engaged in rehabilitation activities and the general public. Their guiding principle is that by regulating who can be a wildlife carer, wildlife and the general public can be protected from unsatisfactory and inappropriate practices, albeit often by well-meaning but unskilled people. 

In Australia, states apply varying levels of scrutiny when vetting candidate carers. For example, in NSW, WIRES, the largest Australian wildlife carer network, requires a prospective carer must have attended their in-house Rescue and Immediate Care Course. This course comprises an online theory component and a one-day practical workshop, and costs AUD$125, which candidates have to fund themselves. The course provides information about the responsibilities of a rehabilitator, and basic rescue and immediate care techniques. Upon successful completion of the course, a written authority is issued allowing the applicant to operate under the terms of the group’s Office of Environmental Health licence. To participate in WIRES training and volunteer with WIRES, the prospective carer must be over 18 years old. Furthermore, for any given species the prospective carers hope to take in, undertaking advanced training in the care of that species is required, along with facilities approval, subject to inspection by an experienced rehabilitator. Specialist courses are available for avian species in general and for raptors, small mammals, macropods, wombats, large mammals, reptiles, flying foxes, microbats, possums, and glider species [79]. 

In the mid-range of this notional scale of regulation implementation, are QLD, SA, TAS, NT, and the ACT, which administer a written questionnaire as part of a candidate’s application to care for wildlife. For example, QLD issues a form that requires details of the length of wildlife caring experience, facilities and training obtained, the available support networks, and the anticipated conservation benefits that will be achieved from caring. If wanting to care for specialist species or genera, candidates must supply the details of two referees, who have to demonstrate their own knowledge and experience and can verify the skills of the applicant [80]. Specialist species and genera include cassowary (*Casuarius casuarius*), emu (*Dromaius novaehollandiae*), echidna (*Tachyglossus aculeatus*), koala (*Phascolarctos cinereus*), platypus (*Ornithorhynchus anatinus*), hawks, eagles, kites and falcons (*Falconiformes*), owls (*Strigiformes*), diving-petrels, storm-petrels (*Procellariiformes*), penguins (*Sphenisciformes*), tropical birds, frigatebirds, cormorants, shags, darters, gannets, boobies and pelicans (*Pelecaniformes*), whales and dolphins (*Cetacea*), bats (*Chiroptera*), dugongs (*Sirenia*), sea turtles (*Cheloniidae*), and snakes (*Serpentes*). In SA, applicants are required to provide detailed information to substantiate their knowledge and experience in the husbandry of the species to be kept [81]. In the ACT, a candidate must become a financial member of a licensed wildlife organisation and then complete basic training to care for wildlife. Under the carers’ code of conduct and code of ethics issued by ACT Wildlife, carers are expected to complete ongoing advanced training, as required, relevant to the species for which they hope to care [82]. 

At the less demanding end of the scale is WA, where potential wildlife carers are merely encouraged to “explore and understand the principles underlying the standards for wildlife rehabilitation in Western Australia” [38] but, since no permit is required to rehabilitate wildlife, this is not mandatory. Licences are issued for periods from one to three years, depending on which state or territory issues them.

In recent years, many Australian states, territories, and wildlife organisations have tried to raise the professional status of carers through educational courses, workshops, and conferences. Although some wildlife carers see this as burdensome, many accept that the complexity of wildlife caring and rehabilitation demands very high standards if the welfare of the animals is not to be compromised. The American state of North Dakota sets such a high standard in this regard that it requires all wildlife rehabilitators to be licensed veterinarians. This has proved prohibitive to the extent that, in 2008, North Dakota had only one remaining wildlife rehabilitator [83]. Washington State requires that permitted wildlife rehabilitator applicants must be a licensed veterinarian or, if not a veterinarian, a wildlife rehabilitator with six months experience in wildlife rehabilitation, with a veterinarian acting as a sponsor to provide guidance [84]. Most other American states require passing an examination as the sole method of demonstrating competency to obtain a rehabilitation licence [85]. By contrast, in the UK, the Royal Society for the Prevention of Cruelty to Animals (RSPCA) has expressed concern that a licence is not currently required to rehabilitate wild animals and accordingly supports the introduction of a system that requires all wildlife rehabilitation centres and rehabilitators to be licensed [86]. The British Wildlife Rehabilitation Council only “encourages rehabilitators to learn about the ecology and behaviour of wildlife and from each other” and publishes a guide for rehabilitators, a similar system to that operating in WA. That said, the need for training is implied in UK legislation. “A person commits an offence if he does not take such steps as are reasonable in all circumstances to ensure the needs of an animal for which they are responsible are met to the extent required by good practice” [87]. An initiative to produce a consensus in Australia on national competency standards for wildlife care is yet to emerge. There is considerable variation in requirements among states and territories (Table 3). 

#### 2.2.1. Inter-State/Territory Differences and Anomalies in Regulations

The main differences and regulation anomalies among states and territories lie in the areas of rescuing, identifying, monitoring post-release, training, and assessment criteria for the ability to release to the wild. For example, in the ACT, the eastern grey kangaroo is designated a controlled animal so, in 2016, annual permits were issued for 11,130 to be culled for commercial reasons, and 1989 adults and 800 pouch young to be culled for conservation reasons [88]. It would seem contradictory, on the one hand, to be killing members of a species and, on the other, rescuing and raising them. Hence regulation exists, in the ACT, which confirms, “licences will not be issued for the hand-rearing of young kangaroos or their release in the ACT” [88] (Section 4.3.1 {f}). Volunteer wildlife carers attending a wildlife rescue in the ACT should know that if they find a pouch young eastern grey kangaroo neonate (joey) they will be expected to euthanase it because it is classed as a non-releasable animal, and “non-releasable animals have a right to euthanasia” [89]. 

A practical solution to this apparent contradiction has been engineered, whereby any rescued eastern grey joeys are taken from the ACT across the NSW border to be raised by carers at Wildcare, Queanbeyan, with the proviso that they cannot be returned to the ACT for release. However, this solution raises other considerations for NSW wildlife carers, because “a person who imports wildlife into New South Wales is guilty of an offence” and “a person who, without authority, liberates a captured protected animal in a place other than the place of its capture is guilty of an offence” [73]. So, an exemption licence is required to allow this border crossing. Because, after rehabilitation, the animal cannot be released back to its place of capture, i.e., in the ACT, the NSW law requires it be euthanased or referred to the Department of Environment, Climate Change, and Water [11]. The ensuing effect on the animal’s welfare as a result of time delays, travel, and changing environments could be considerable, just as the effects on the moral and mental well-being of wildlife carers could be significant. 

The identification of animals is critical for the analysis of projected needs and welfare outcomes when seeking to monitor the progress of native fauna through the rescue, rehabilitation, and release procedure. None of the states or territories require a rescued animal to be individually identifiable. Indeed, the opposite is the case in VIC, WA, and QLD, where marking an animal is discouraged and those who do so are guilty of an offence. The Victorian Wildlife Act, 1975 (VIC), part v11 section 51 states: “any person who marks protected wildlife by means of a ring, band, dye, or other means whatsoever without the authority in writing of the secretary shall be guilty of an offence against this act” [31]. The Western Australian Standards for Wildlife Rehabilitation 2015 (WA) Chapter 2 (Regulation 28A) states: “if fauna are marked in any way the rehabilitator is no longer ‘caring for sick or injured fauna’ but is conducting research” [38]. Also, in section 57 of the Wildlife Conservation Regulations (WA) 1970: “a current licence is required from the department to mark fauna for identifying purposes post release” [37]. In QLD, the Nature Conservation Act 1992 (Qld) section 15.2.10, states that, “tagging, banding, or other marking, including microchip or passive integrated transponder (PIT) implanting must only be performed as part of a Department of Environment and Science approved program” [17]. NSW takes a somewhat different approach and actively encourages the marking of rehabilitated wildlife, albeit not until the time of release of the animal. The NSW Code of Practice for injured, sick, and orphaned protected fauna 2011 (NSW) Section 12.3.1.4. states: “fauna rehabilitators should arrange for fauna to be tagged, banded, and micro-chipped or marked as appropriate for individual identification prior to release” [9]. TAS and NT are the only jurisdictions in which marking or labelling a rescued animal may be required by the relevant authority upon acquisition by the rehabilitator. In TAS, the Nature Conservation Act 2002 (TAS) Part 2 section 17 states: “the Secretary may direct the holder of a licence to mark or tag wildlife in the holder’s possession” [26]. In the NT, the Territory Parks and Wildlife Conservation Act 2014 (NT) Pt 1V Division 6. 57. (1) (b) v, states: “authority may require the labelling or applying of markings to the animal” [15]. 

There are lengthy descriptions in all the state and territory regulations about how and when inspectors can examine rescued animals and their records, and how all the different permits operate. So, it is surprising to note that none of the states or territories require any rescued animal to be individually identifiable. This abiding lack of identification negates the ability of any third party’s inspection of a carer’s practice to be reliable. Conceivably, for any record being kept, an animal that escapes, dies, or is even given away can easily be substituted with another. An animal that is inappropriately returned to the wild and then causes damage or injury cannot be traced back to the person who released it. This renders unsustainable any investigation of the offence of releasing an animal without a permit or any claim for damages. These seem to represent compelling reasons for identification being required by regulation. 

An illustrative case of human wildlife-related injury from TAS is one of a wombat (*Vombatus ursinus*) attacking people and causing injuries that resulted in their needing hospital treatment [90]. The suspicion was that the wombat had been hand-reared and was not able to cope after being released to the wild. The wombat was captured and euthanased by the Tasmanian Parks and Wildlife service. Had it been identifiable by a microchip or tag, the outcome could have been different for the animal, as well as for the person who may have released it [90]. Had its history been known, the wombat may have been suitable for captive housing in a wildlife park and a life in captivity, and the carer identified and appraised in rearing-and-release protocols. 

Similarly, if rehabilitated wildlife that was returned to the wild were identified by microchip, all roadkill and other casualties could be scanned to reveal if they were originally injured animals or ones that had been hand-reared. This would provide a valuable source of data for research into survival rates. 

#### 2.2.2. Intra-State/Territory Differences and Anomalies in Regulations

Beyond the apparent inter-state/territory contradictions in legislations described above, there are similar apparent contradictions and anomalies in intra-state legislation. One example concerns the following aspect of QLD legislation: “tagging, banding, or other marking, including microchip or passive integrated transponder (PIT) implanting must only be performed as part of a Department of Environment and Science approved program” [91] (Section 15.2.10). This provision seems to be contradicted by two others, namely that “a register must be kept by each wildlife rehabilitator for all protected animals rescued or cared for including identifying number or name” [19] (Section 16.2.1. 2.), and “to gauge the effectiveness of various rehabilitation and release techniques, post-release sightings of known rehabilitated wildlife should be recorded and kept” [19] (Section 16.3.2.). 

A further example of contradictions in intra-state legislation appears in Victorian legislation, stipulating that, “The release site should be suitable habitat in the general facility from which the animal was originally collected. For instance, if the animal were found injured on a highway, an area of bushland adjacent to the highway would be a suitable release site” [33] (Case assessment, bullet point 15), but requiring that “the factors that lead to the original injury or condition must not pose an unacceptable risk to the animal upon release’ [33] (Case assessment, bullet point 18). The suggested example of using an area of bushland adjacent to the highway to release the animal where it was injured would surely present an unacceptable risk of a WVC.

### 2.3. Standards of Care, Facility Requirements, and Identifying of Animals

All Australian states have issued either a code of practice, general guidelines, requirements, or standards for the rehabilitation of wildlife. For the territories, the NT has individual guides for caring for animals and the ACT is in the process of developing a code of practice. A draft code of practice for the Welfare of Native Wildlife—Rescue, Rehabilitation, and Release is currently with the ACT Animal Welfare Advisory Committee [92]. 

In general, the purpose of these codes is to benchmark standards to provide the minimum acceptable animal welfare and conservation outcomes. Notably, all states and territories stipulate that all costs involved in providing wildlife rehabilitation are to be met by the wildlife carers (Table 4). 

### 2.4. Funding and Monitoring of Carers/Carer Networks

All states and territories require at least some record-keeping by wildlife carers, but the complexity of the records required varies. Four states—NSW, QLD, TAS, and WA—specify very comprehensive standards and guidelines for what records are required from wildlife carers in their codes of practice. VIC and SA require that records, pertinent to the taking and release of the animal, are maintained, but wildlife rehabilitators are only “encouraged to keep their own additional details regarding the care, treatment, and release of the animals” in VIC [94] whereas in SA, a record book is provided with a requirement for “records to be provided in accordance with the prescribed process on the permit” [23]. The NT requires a wildlife report and an application for release form to be completed by wildlife carers, but requires no details of the care provided during rehabilitation [14]. The ACT requires carers to keep records and submit returns annually [95]. 

NSW requires that “records must be submitted to the Wildlife Licensing and Management Unit of the Office of Environment and Heritage in an approved electronic format on an annual basis” [9] (Section 14. 1.1. para 7. 201). VIC lists key record-keeping obligations, including the need to “submit a completed return form by no later than 14 April detailing wildlife in your possession”, and also supplies a record-book in which “all wildlife transactions must be recorded” [94]. However, this does not include details of the care or health status of the animals under rehabilitation. In Tasmania, wildlife carers are strongly encouraged to keep records for all animals in rehabilitation, and there is a requirement under the Wildlife (General) Regulations 2010 (TAS) section 30 for record-keeping and for providing a return of the records for inspection, when requested [27]. SA requires returns to be made annually by 30 June [22] (Schedule 5). Other states require similar records to be kept in a manner that can be readily examined, analysed, and clearly understood and made available to officials on request. Little state or territory funding is supplied to wildlife carers to meet the costs of providing these records (Table 5). All states legislate to enable officials to require wildlife carers to submit to inspection of their records, premises, or facilities they provide for rescued rehabilitating animals (Table 5).

### 2.5. Pre- and Post-Release Preparations, Behaviour Modification, Protocols to Be Followed, and Monitoring of Rehabilitated Wildlife Being Returned to the Wild

Depending on the species, the rescuing, rearing, and releasing of native animals to the wild can take up to two years. Before releasing an animal, an assessment can evaluate whether an animal has developed maladaptive behaviour. It can also evaluate whether an animal has learned behaviours appropriate to it surviving in the wild, so that its well-being will not be compromised. Post-release monitoring can validate the accuracy of the assessment.

#### 2.5.1. Pre-Release Behavioural Advice

Advice is given to wildlife carers in all jurisdictions to avoid desensitising animals undergoing rehabilitation, i.e., treating them as a pet or member of the family and allowing exposure to humans (Table 6). It describes methods that may prevent this happening. Also, advice is given on how to prevent animals becoming desensitised to those stimuli that may require them to show a flight or fight response to survive, when released. Further advice is supplied about allowing animals to learn behaviours to equip them for life in the wild. Summarised from the codes of practice and regulations, Table 6 provides the type of advice relating to pre-release behaviour methodology on a state/territory basis. None of the codes or guidelines mention the provision of behavioural conditioning prior to release, via a behaviour modification programme, to sensitise and condition an animal to relevant environmental hazards, such as road traffic, predators, and humans. The ACT has no code of practice to offer advice, although one is under preparation [92].

#### 2.5.2. Protocols for Releasing or Not Releasing Rehabilitated Native Fauna to the Wild

All states and territories declare the intention that all rehabilitated wildlife is returned to the wild, (Table 7). NSW, SA, and NT require a permit to be obtained to release an animal. TAS requires notification of the Department of Primary Industries, Parks, Water, and Environment prior to release. QLD, NSW, and VIC stipulate release but only if wildlife is assessed as being physically and behaviourally fit (Table 7). VIC stipulates release within 24 h of an animal being ready for release. WA states that release must be as soon as practicable after the animal recovers or becomes capable of fending for itself. The ACT requires rehabilitated animals to be returned to the wild but stipulates no time limit on holding suitable animals prior to release.

#### 2.5.3. Suitability of Rehabilitated Animals for Release

Assessment of the suitability for a rehabilitated animal to be released varies considerably among the states and territories. NSW and QLD require an assessment by a wildlife veterinarian or an experienced rehabilitator, while VIC requires an assessment by a veterinarian or experienced wildlife rehabilitator. No definition is given to explain the difference between a veterinarian and a wildlife veterinarian or what constitutes an experienced wildlife rehabilitator. TAS requires a declaration of the process that will be used for the release of protected wildlife to be submitted to and approved by the conservation branch of the Department of Primary Industries, Parks, Water, and Environment. WA has adopted a self-regulated approach, with advice on the suitability of rehabilitated animals for release being given to wildlife carers. SA does not require an inspection but, significantly, states that: “the release of long-term captive animals is rarely justified on conservation or animal welfare grounds” [101]. The NT and ACT require no assessment of the suitability of rehabilitated animals for release. None of the states discuss or give protocols necessary for an objective behavioural assessment of such suitability, how it has to be undertaken, or where the necessary assessment facilities should be located, nor is there any mention of how assessors should be remunerated for their work. 

Assessment of the suitability of rehabilitated animals to return to the wild can involve the assessors making life-or-death decisions for the animals, in some states. Both the animals’ health and behavioural suitability for release need assessment (Table 8). In a 2012 survey of current rehabilitation practices in Australia, Guy and Banks [102] observed that there were no consistent criteria for the suitability of an animal for release, although as early as 1989, Kleiman proposed five behavioural attributes as a minimum required for animals to survive in the wild after rehabilitation [103]. These were the ability to avoid predators, acquire and process food, interact with conspecifics, construct and discover shelter sites or nest sites, and to navigate through its natural habitat. Additionally, Kleiman added that to “achieve a wild state, an animal must show a fear and avoidance of humans”. All states and the NT stipulate that some criteria, similar to those behavioural attributes in the above list, must be met, but the details differ considerably among jurisdictions [9,14,19,23,28,38,104], as summarised in (Table 8). Evidence of the need to adopt behavioural attributes is suggested from the analysis of survival rates of Tasmanian devils (*Sarcophilus harrisii*) recently released to the wild during a rewilding project [105]. The longer the period in captivity, the higher the mortality when released, particularly from WVCs. This suggests that a lifetime of habituation towards humans and vehicles, as a result of daily exposure in captive facilities, compromised the Tasmanian devils’ vehicle avoidance skills. In their analysis of survival rates, Grueber et al. concluded: “Our results imply that long-term captive breeding programs may produce animals that are naïve to the risks of the post-release environment. Growing evidence suggests that behavioural and genetic changes mediated by a captive-rearing environment may negatively impact the suitability of captive animals for release” [105].

The complexity and detail of the above standards mean that it is unlikely that, regardless of their experience, lay rehabilitators would be qualified to assess the health requirements in Table 9. If these are to be met, it should be mandated that all animals be examined by a veterinarian, qualified in wildlife medicine, before release. Undertaking behavioural assessments that could lead to either euthanasia or the inappropriate release of an animal requires considerable expertise and facilities and the need for a thorough knowledge of the species’ wild behaviour [106]. The Australian regulations concerning the release of rehabilitated wildlife seem to assume that a veterinarian or experienced wildlife carer has these competences. This appears to be a flawed assumption when one considers that the training time allocated to animal behaviour and veterinary behavioural medicine during the training period to become qualified as a veterinarian is constrained. 

It is one matter to set behaviour standards, but the practicality of complying with these assessment standards could be time-consuming and difficult to implement, and, for some species, require large infrastructure and technical facilities. Items 15 and 16 in the behaviour assessment (Table 9) state: “an animal must not be attracted to humans or sights, sounds, or smells that are specific to captivity” and “must show a fight or flight response”. Legislated animal behaviour traits cannot be objectively assessed unless appropriate facilities are provided. Reintroduction biology is an emerging field of science [103]. However, the release of thousands of hand-reared native animals back to the wild through the wildlife carer networks [62] provides research opportunities for reintroduction research that includes experimental studies. 

#### 2.5.4. Action on Non-Releasable, Rehabilitated Wildlife

If, during the rescue, rehabilitation, or assessment prior to release, an animal is deemed to be non-releasable, the protocols to be followed vary from mandatory euthanasia to being retained in captivity. In VIC, “non-releasable” equates to euthanasia. The Wildlife Shelter and Foster Carer Authorisation Code 2018 states, “you must euthanise wildlife that cannot be released” [100] (condition 22). The other jurisdictions demonstrate more flexibility. For example, NSW states the Department of Environment, Climate Change, and Water will assess whether a rehabilitator or others could hold un-releasable animals permanently in captivity, e.g., if the animal will serve as a companion in a social group, or will be involved in a recognised education program, or will be involved in scientific research [11]. If the above is not possible, the animal must be euthanased. In SA, it is possible to obtain a permit to keep rehabilitated wildlife and to buy and sell wildlife [21] (Schedule 2, Section 3). However, if a reasonable expectation of a high quality-of-life is not available then the animal must be euthanased. Neither the timescale for assessing satisfactory expectations nor the methodology to obtain them are specified. The ACT allows the keeping of a non-releasable animal under licence as long as it was not taken from the wild, which would be an offence under the Nature Conservation Act [5]. The NT permits the keeping of wildlife as pets under licence, but advise that if the animal is unsuitable for release and has a poor chance of survival in the wild then it should be humanely euthanased [14] (option 1.2.). QLD has a mechanism whereby a non-releasable animal can be referred for placement through the Queensland Species Management Plan and, if not, must be euthanased [91] (Section 12.3.2.). WA regulates that wildlife carers cannot keep non-releasable animals and must surrender them to a wildlife officer or, with permission from the Minister for Agriculture and Food, give them to a person who is licensed to keep them. It states that, if it is unlikely that the animal cannot be fully rehabilitated, euthanasia should always be considered the preferred option [38] (Chapter 5 paragraph 3). In the ACT, Jones comments that, “we strongly discourage the keeping of any non-releasable wildlife by carers and have thus far been successful in finding new homes for these creatures in wildlife parks, etc.” [95].

#### 2.5.5. Release Methodology

Post-release behavioural advice is aimed at using the most likely method to secure a successful release. However, post-release monitoring is minimal (Table 9). Most jurisdictions recommend either a hard release or soft release and that, either way, the animals should be returned to the location where they were found, if possible. A hard release is generally defined as one where the animal is released without any support, whereas a soft release is where an animal is provided with temporary post-release support, which may include supplementary feeding, shelter provision, or protection from predators. There is consensus that the sooner an animal is returned to the wild the better. NSW is the only jurisdiction to actively encourage post-release monitoring. “Fauna rehabilitators should arrange for fauna to be tagged, banded, and microchipped or marked for individual identification prior to release. Fauna rehabilitation groups and zoological parks are encouraged to participate in post-release monitoring programs to determine survivorship” [9] (Section 12.3.1.4.). In WA, the Standards for Wildlife Rehabilitation in Western Australia state to “monitor post-release if possible” [38] (Chapter 1, {9}). 

### 2.6. To Release or Not Release Rehabilitated Injured and Orphaned Native Animals to the Wild

In general, all Australian jurisdictions require that rehabilitated animals be returned to the wild. No distinction is made between those who are injured and those who have been orphaned and will require hand-rearing, and thus will spend a considerable time in rehabilitation. The consequences of this lack of distinction is examined.

#### 2.6.1. Returning Rehabilitated Injured Animals to the Wild

Adult and juvenile native animals raised in the wild have all their innate and learned behaviour instincts intact when they are injured and rescued. Unless they remain in captivity for a prolonged period or are subjected to inappropriate housing and handling, these behaviour patterns will persist and become operative once released. As long as release protocols are followed (Table 9), the animal will have an opportunity to survive and a life equally worth living as that which would have been expected prior to the injury. From a conservation perspective, there is also another advantage to returning a rehabilitated, previously injured, animal to the wild. Learning theory would suggest that an animal subjected to a traumatic event, such as being struck by a vehicle, and able to pair this with a salient stimulus, such as the sound or headlights of a vehicle, can remember this after only one incident [108]. The animal learns to avoid the aversive stimulus [109]. Similarly, it was shown that avoidance behaviour can be extremely persistent [110]. Also, there is evidence of aversive sign-tracking systems, in which animals’ tendencies to withdraw are determined by Pavlovian contingencies, which render them reliable signals for the occurrence of an aversive stimulus, such as a WVC [111].

The significance of this is that an animal who has been injured by a road vehicle could remember the sound, sight, or even odour that pre-empted the impact with the vehicle. When returned to the wild and encountering salient visual, olfactory, or auditory signals, the animal is likely to show a flight avoidance response and hence boost its chances of survival. A female could pass this behaviour to its offspring through association with avoidance responses and enhance their chances of survival. Innate behaviour is the result of the interaction of the species with its environment during evolution, and success at staying alive leads to coded information in the genome [112]. In a gregarious species, such as a kangaroo, an individual survivor might well become the animal that passes this behaviour to the rest of its mob. When a critical number of animals have survived such encounters with vehicles and have learned avoidance, then over multiple generations the avoidance behaviour may eventually become an innate behaviour similar to the way that, after being hunted during the last 50,000 years, kangaroos became innately wary of humans. 

#### 2.6.2. Releasing Hand-Reared Orphan Animals to the Wild

Requiring that hand-reared orphans must be returned to the wild may be difficult to defend on conservation, ethical, moral, and practical grounds. Indeed, the effectiveness of releasing hand-reared animals to the wild for conservation reasons is questioned by many authorities both in Australia and worldwide. VIC and SA state that rehabilitation has limited benefits for biodiversity conservation [23,33], and that releasing long-term captive animals is rarely justified on conservation or animal welfare grounds [16]. In an example specific to the ACT, it is stated that, “there is no justification for the hand-rearing and release on conservation grounds, as the eastern grey kangaroo is an abundant species” [2]. In the UK, orphans who have been hand-reared are released back into the wild [113]. However, Kelly et al. state, “such releases receive very little attention from conservation biologists as they are seen to have little conservation value” [114]. This may be because the most numerous species that are hand-reared in the UK, such as badgers (*Taxidea taxus*) and fox (*Vulpes vulpes*) cubs, deer (*Cervidae cervinae*), squirrels (*Sciuridae*), and hares (*Lepus Europaeus*) are well represented in the wild [113] and the reintroduction of hand-reared orphans into the wild has limited success [115,116,117,118].

The consequences of returning hand-reared animals to the wild are not confined to their own generation. For example, there may be behavioural aspects to the release of hand-reared orphan animals that may actually compromise conservation by associative learning and the subsequent social transmission of conditioned responses. This runs counter to the positive effects of rehabilitating and releasing injured animals mentioned previously. For example, consider a female marsupial roadkill victim that did not react to a motor vehicle threat and get out of the way quickly enough. This might be because of a diminished fear or startle response to a stimulus or an innate slower reaction time. If an orphan is rescued from this female’s pouch, hand-reared, and released to the wild, the high-risk behaviour of the mother may have been vertically transmitted to her offspring, thereby making the released orphan itself more likely to become a roadkill victim. It could be argued that releasing hand-reared orphans to the wild over several generations inadvertently selects for roadkill. 

### 2.7. The Mental and Physical Well-Being of Rehabilitated Native Animals Released to the Wild

Contemporary animal welfare practice has evolved from just the physical provisions included in the Five Freedoms [119] to the need to consider a life worth living [120,121,122]. It has been argued that a new moral framework is needed that connects the treatment of animals more directly to fundamental principles of liberal-democratic justice and human rights [123]. These encompass the mental as well as the physical well-being of animals. Mental well-being encompasses the mental and emotional state of an animal [124,125,126]. This concept requires that animals should feel well mentally and should not be subjected to excessive negative emotions, such as fear, stress, pain, and hunger. In addition to avoiding negative emotions, animals should be able to experience positive emotions in the forms of pleasure or contentment if they are to have a life worth living, for example, by being able to perform important, normal behaviours, such as rest, play, or social interactions with conspecifics. Ethologists accept that animals have feelings [127,128,129,130] and thus it can be expected that hand-reared animals released to the wild could suffer.

#### 2.7.1. Post-Release Behavioural Effects of Habituation or Desensitisation to Humans Prior to Their Release, on Hand-Reared Rehabilitated Animals

Australian marsupial pouch young (joeys) account for most of the orphans that require hand-rearing and release, but there are also birds (*Aves*) and bats (*Chiroptera*). The ability to rear chicks successfully to the time of release and survival in the wild has been demonstrated in several conservation projects, such as saving the Californian condor (*Gymnogyps californianus*) [131], and the whooping crane (*Grus Americana*) [132]. By avoiding human contact, wearing costumes that resembled adult birds and feeding using ‘puppet heads’ or artificial bills, any possibility of imprinting, habituation, or desensitisation to humans was successfully avoided. Similarly, with hand-reared bats (*Chiroptera*), it is possible to rear orphans without imprinting them on humans. Specifically, as demonstrated with pipistrelle bats (*Pipistrellus* spp.), as long as there is extensive pre-release conditioning of flight training in a large flight cage, release to the wild is not characterised by reduced survivorship [114]. Thus, there is no moral or ethical reason to believe the welfare of hand-reared birds or bats is compromised when released to the wild. 

In contrast, when hand-rearing juvenile mammals (joeys) that require milk substitute feeding from a bottle it is difficult, if not impossible, to avoid habituating them to the olfactory, auditory, and visual stimuli of human presence and the human environment. A joey in a cloth pouch hanging from a hook in a kitchen does not experience the smells of a female’s pouch, the sounds of the bush, or the natural movements of a mother. Indeed, the likelihood of the joey associating anthropogenic stimuli with food and comfort seems considerable. As it matures, the joey may well experience the interior of a vehicle when travelling to be examined by a veterinarian, or hear the sounds of a neighbour’s dog barking, or other auditory and olfactory attributes of urban existence. All of these experiences may extend the joey’s desensitisation to the human environment and to stimuli, that ordinarily would produce a fear/flight response when released, if the chance for survival is to be optimised. Taking all of this together, the possibility that hand-rearing rescued offspring of wild animals and releasing them to the wild through a hard, soft, or managed release operation leads to a life worth living and the ability to exhibit normal behaviour seems remote. Under these circumstances, the normal behaviour for such animals is the behaviour they have learned in captivity. Many wildlife carers may equate releasing an animal and seeing it disappear into its natural habitat with success but, without post-release monitoring, this may be an unfortunate convenient illusion. The mental state of the released animal may not be the happy state that carers may prefer to assume.

#### 2.7.2. Assessment of Likely Mental and Emotional State of Rehabilitated Animals Released to the Wild

To comment on an animal’s welfare, one must be able to assess objectively whether it is compromised by its mental and emotional state. Although there is the danger that the use of a definition based on human experience and feelings will increase the myopia that already exists when considering animal welfare, the definitions stated by Broom [133] and Hemsworth et al. [134] define animal welfare appropriately, as “the state of an individual animal as regards its attempts to cope with its environment” and as “a state within the animal, and a simplistic definition might be how the animal feels now”. The common human intuitive reaction that placing rehabilitated animals into the wild is the natural approach to take may not consider what the animal feels. In defining ‘naturalness’, Yeates concludes that “the vague assumptions that naturalness is reliably associated with better well-being are unfounded” [121]. Australia is set to follow Europe, New Zealand, and Canada in recognising, for the first time in animal welfare legislation, that animals have sentience, i.e., the ability to have feelings such as pain and, pleasure and the ability to suffer. The ACT is leading other states and territories in introducing this concept in the Animal Welfare Legislation Amendment Bill (ACT) 2019 [72].

The basic question for hand-reared animals is what price they will have to pay to try to survive once released. Before releasing a hand-reared animal to the wild, it is appropriate to assess indicators of its likely mental, emotional, behavioural, and cognitive state after release. Socialisation and learning to live in an environment are vital for the survival of all animals. When striving to obtain and secure food, prey species are particularly exposed to agonistic behaviour from other animals motivated to defend themselves. If predator species have not learned how to react to auditory, olfactory, and visual stimuli of conspecifics, when confronted, their chances of survival are diminished. Similarly, with no cognitive map of the habitat into which they have been released to the wild, naïve orphans could go through a period of fear and stress, as their normal behaviour is no longer appropriate to surviving in their new environment. 

Resource holding potential is a term describing the motivation and capacity an individual has to continue to fight, work, or endure [135,136]. It can be estimated as a function of the motivation to obtain a resource, what needs to be done to acquire the resource, and what it will cost obtaining and defending that resource. The number of days released animals remain in a fearful state before their resource holding potential is adequate, and the level of pain from hunger or thirst sufficient to motivate them to overcome their fear of a novel environment, are difficult to estimate. 

#### 2.7.3. Practical Issues of Undertaking Mental and Physical Assessments of Released Rehabilitated Animals

To understand whether the mental and physical well-being of a released animal are being compromised would require the tracking of all released animals over an extended period and a reliable system for identifying them. The financial cost of meeting such a tracking requirement would be considerable, with suitable tracking devices costing over $1000 each and, furthermore, it would be unlikely to obtain ethics committee approval [137]. In contrast, identifying each animal by means of a microchip, leg-band, or tag could cost as little as $20/animal, and so would be a practical proposition. 

#### 2.7.4. Alternatives to Releasing Rehabilitated Animals to the Wild

If it is considered that for conservation, ethical, moral, and practical reasons, rehabilitated, hand-reared wildlife should not be placed into the wild, then an alternative is required. Available alternatives include euthanasia or lifelong captivity. If it is possible to provide these animals with a life worth living in captivity, this may represent a workable solution. This idea already has a degree of acceptance. The National Parks and Wildlife Service Rehabilitation of Fauna Policy 2010 (NSW 2010) states: “The NPWS will consider on its merits any application from a zoo or fauna park licensed under the Exhibited Animals Protection Act 1986, to recruit protected fauna which has been hand-raised or is undergoing rehabilitation into the exhibition stock holdings of that park. Approval for the acquisition or retention of such an animal will be subject to the concurrent approval of the Registrar of the Exhibited Animals Protection Act.”

With the exception of VIC and WA (Table 6), carers or institutions are permitted to keep wildlife under licence. The idea of keeping wildlife as pets has been explored by a feasibility study featuring conservation, welfare, and industry metrics [138]. This study advocated a conservation-through-sustainable-use strategy and placing a monetary value on native animals as companions to contribute to their protection in the wild. The study also says that replacing some non-native companion animals, such as cats, with natives could also have conservation benefits. A separate report by Hopwood also argued that the Government had erred by preventing people from keeping some species of Australian fauna as pets [139]. Enriched enclosures can be built to provide wildlife with an area appropriate to their behavioural needs. Examples of how wildlife can be kept in captivity in natural surroundings and enjoy a life worth living include Mulligans Flats in the ACT, Australian Reptile Park’s ‘Devil Ark’ facilities at Barrington Tops in NSW, and the Devil Island Project’s free-range enclosure facilities in TAS. 

Many wildlife rehabilitators equate release with success, but very few post-release survival studies have been conducted to support or, for that matter, challenge this view. Those that have been conducted present a largely unsuccessful report, with the notable exception of wombats released onto private property [69]. The attempt to re-introduce Eastern Quolls (n = 20) on the Australian mainland in 2018 met with an over 70% death rate in three months due to vehicles, natural predators, and foxes [140]. A similar fate befell Tasmanian devils (n = 39) released on Tasmania’s Forrester Peninsula, with a 25% mortality within the first weeks of release [141]. It is important to consider how wildlife carers might grieve or otherwise cope if they knew for certain that 70% of the animals they release die within three months [62]. 

## 3. Conclusions

This article has reviewed and critiqued the systems that currently regulate the care of Australian native animals that are rescued, rehabilitated, and released. It has found that the current systems are far from perfect, rely on many assumptions, are riddled with inconsistencies, and condone practices that may compromise the environment and the welfare of wildlife carers. The systems have evolved this way because of the influence of the state-by-state approach to policy development that prevails in Australia. Also, because the subject matter is complicated, it may have been left to policy-makers who may have been ill-equipped to craft appropriately reflective regulation. This is unsurprising, given that animal law as a discipline has been emerging only since 2010 in Australia and, in Australian tertiary education, currently exists as the inclusion of only one or two elective units in legal degrees. Many aspects of relevant policy rely on assumptions that are not based on scientific evidence. Vague assumptions that naturalness in releasing animals to the wild is reliably associated with better well-being are unfounded [121]. This review and critique confirms that such assumptions are often wrong. It indicates the need for an evidence-driven approach to wildlife rehabilitation. Such an approach is problematic when legislation and regulatory systems are fragmented, contradictive, unenforceable or unenforced, and thwart the possibility of data collection. 

A reformed national initiative could resolve many of the current difficulties that face care-givers/rehabilitators to the many and varied Australian native animals that would otherwise perish or require euthanasia. It is hoped that such a national initiative and review would result in the preparation of nationally consistent science-based, best practice guidelines for native animals in care and beyond. This could provide a significant boost to the well-being of both carers and native animals. The current regulatory controls are heavily weighted towards the rescue and rearing of the animals. However, the release of the animals to the wild raises concern for their post-release well-being. It is essential that all rescued animals that are to be released to the wild are reliably identifiable. Based on the existing regulations, expert assessment of animals’ suitability for release is also required. Unless these criteria are met, releasing hand-reared wildlife to the wild should be discontinued, and other options, including allowing the keeping of some native animals and the use of large-scale facilities, such as national parks, islands, and fenced enclosures, explored. 

Regulatory frameworks need to balance the needs of rescued wildlife, wildlife carers, and conservation. The public and wildlife carers need to be confident that regulation is consistent among jurisdictions and reflective of best practice for the rescued wildlife and the environment. The protection of Australian injured or orphaned native wildlife should be recognised as an important animal welfare issue. 

## Figures and Tables

**Table 1:** Australian state and territory animal legislation, regulation, and codes of practice/policy.

State/Territory	Legislation	Regulations	Codes of Practice/Policy
Australian Capital Territory (ACT)	Animal Welfare Act 1992 (ACT) [1]	Nature Conservation Regulation 2015 (ACT) [2]	Code of Practice for the Welfare of Captive Birds 1995 (ACT) [3]
			Reptile Policy 2018 (ACT) [4]
	Nature Conservation Act 2014 (ACT) [5]		
			Code of Practice for the Welfare of Amphibians in Captivity 2004 (ACT) [6]
New South Wales (NSW)	Prevention of Cruelty to Animals Act 1979 (NSW) [7]	Prevention of Cruelty to Animals Regulation 2012 (NSW) [8]	Code of practice for injured, sick and orphaned protected fauna 2011 (NSW) [9]
	Biodiversity Conservation Act 2016 (NSW) [10]		The Rehabilitation of protected fauna policy 2010 [11]
Northern Territory (NT)	Animal Welfare Act 2017 (NT) [12]	Animal Welfare Regulations 2013 (NT) [13]	Guide for Caring for Wildlife 2018 (NT) [14]
	Territory Parks and Wildlife Conservation Act 2014 (NT) [15]		
	Animal Protection Bill 2018 (NT) Pending [16]		
Queensland (QLD)	Nature Conservation Act 1992 (QLD) [17]	Animal Care and Protection Regulation 2012 (Qld) [18]	Code of Practice Care of Sick, Injured or Orphaned Protected Animals in Queensland 2013 (Qld) [19]
	Animal Care and Protection Act 2001 (Qld) [20]		
South Australia (SA)	National Parks and Wildlife Act 1972 (SA) [21]	National Parks and Wildlife (Wildlife) Regulations 2016 (SA) [22]	General Guidelines for the Management of Protected Wildlife in Captivity in South Australia 2010 (SA) [23]
	Animal welfare Act 1985 (SA) [24]		Guidelines on Taking from the Wild (SA) [25]
Tasmania (TAS)	Nature Conservation Act 2002 (TAS) [26]	Wildlife Regulations 1999 (TAS) [27]	General Requirements for the Care and Rehabilitation of Injured and Orphaned Wildlife in Tasmania, 2008 (TAS) [28]
	Threatened Species Protection Act 1995 (TAS) [29]		
	Animal welfare Act 1993 (TAS) [30]		
Victoria (VIC)	Wildlife Act 1975 (VIC) [31]	Wildlife Regulations 2002 (VIC) [32]	Code of Practice for the Welfare of Wildlife during Rehabilitation 2017 (VIC) [33]
	Prevention of Cruelty to Animals Act 1986 (VIC) [34]	Prevention of Cruelty to Animals Regulations 2008 (VIC) [35]	
Western Australia (WA)	Biodiversity Conservation Act 2016 (WA) [36]	Wildlife Conservation Regulations 1970 (WA) [37]	Standards for wildlife rehabilitation in Western Australia 2014 (WA) [38]
	Animal Welfare Act 2002 (WA) [39]		

**Table 2:** Taking of Injured and Orphaned Wildlife.

State/Territory	Assisting with Dead or Injured Wildlife	Requirement for Subsequent Action	Possess an Animal
ACT	Animal Welfare Act 1992 (ACT) [1] (Pt 2 10 {1}) Nature Conservation Regulation 2015 (ACT). [2] (Section 8 s 269) Must assist.	Animal Welfare Act 1992 (ACT) [1] (Pt 2 10 {2}) Yes, within 72 h	Nature Conservation Act 2014 (ACT) [5] (Chapter 6 Pt 6 Div. 6.1.2. Sect 132) Nature Conservation Act 2014 (ACT) [5] (Chapter 11 Pt 11.1) Licences to keep, sell, import into, and export out of the ACT are required for all animals (dead or alive, whole or parts of) except those scheduled as exempt species under the Act.
NSW	Biodiversity Conservation Act 2016 (NSW) [10] (No 63. Part 2.1) Only if licensed.	Code of Practice for Sick and Orphaned Protected Wildlife (NSW) [9] (4.1.2) Assessment within 24 h required. Biodiversity Conservation Regulation 2017 [NSW] [73] Notification of possession within 3 days.	Biodiversity Conservation Act 2016. [10] (Part 2. 2.5 {1} {2}) A person who possesses or attempts to possess a protected animal is guilty of an offence.
NT	Territory Parks and Wildlife Conservation Act 2014 (NT) [15] (Division 8 {1}) A person must not take or interfere with protected wildlife unless authorised to do so as in the rescue of an animal.	Not required.	Territory Parks and Wildlife Conservation Act 2014 (NT) [15] (Division 6. 55 (c) & 62) Yes, can keep protected wildlife, and can bring protected wildlife into, release or take out of the Territory. Permit required. Wildlife becomes the property of the holder of the permit. 1237 permits were issued in 2017 to keep native protected wildlife.
QLD	Nature Conservation (Wildlife Management) Regulation 2006 (Qld) [74] (Section 59 {4}) Rescuer of animal must surrender the animal to a licensed rehabilitator or conservation officer.	Nature Conservation (Wildlife Management) Regulation 2006 (Qld) [74] (Section 59 {4}) Within 72 h.	Nature Conservation Act 1992 (Qld) [17] (Part 5 Division 3. 83) Yes. An animal ceases to be the property of the State if the animal is taken under a licence or permit.
SA	Animal Welfare Act 1985. (SA) [24] (Pt3. 15A) Duty of person in charge of a vehicle in case of accidents involving animals. Must inform the owner or an inspector of the accident occurring.	Animal Welfare Act 1985 (SA) [24] (Pt3. 15A) Within 24 h	National Parks and Wildlife Act 1972 (SA) [21] (Sect. 51 Taking of protected animals {1}) Subject to this part, a person must not take a protected animal or the eggs of a protected animal.
TAS	No requirement to stop or report.	General Requirements for the Care and Rehabilitation of Injured and Orphaned Wildlife in Tasmania 2012 (TAS) [28] Report at the earliest opportunity on the first day of business after receiving the animal.	Wildlife Regulations 1999. (TAS) [27] (Part 2. Section 16) Except as may be authorised by a permit, a person must not take, buy, sell or have possession of any form of specially protected wildlife or the products of such wildlife.
VIC	Road Safety Act 1986 (Vic) [75] (Section 61) If an accident occurs whereby any person is injured or any property (including any animal) is damaged or destroyed, the driver of the motor vehicle—(a) must immediately stop the motor vehicle; and (b) must immediately render such assistance as he or she can.	Code of Practice for the Welfare of Wildlife during Rehabilitation 2017 (Vic) [33] Animals must be assessed accurately and without delay.	Wildlife Act 1975 (Vic) [31] (Part 111A 28 A {1} and {F})The Secretary may give written authorisation to a person to take wildlife for the purposes of enabling the care, treatment or rehabilitation of sick, injured or orphaned wildlife.
WA	Reporting a Traffic Crash 2018 [76] No requirement to stop, assist or report.	None	Anyone can rehabilitate wildlife without a permit.

**Table 3:** Australian licensing, permitting, and training requirements for wildlife carers to keep wildlife.

State/Territory	Permit/License Required	Demonstration of Competence	Permit Renewal
ACT	Nature Conservation Act 2014 (ACT) Required. Must be a member of a wildlife organisation and reside in the ACT [5] (Chapter 6 Division 6.1.4) Licences will not be issued for the hand-rearing of young kangaroos or their release in the ACT [88]	Minimum requirement is to have attended an orientation course and basic bird care course. Carers are expected to complete ongoing advanced training, as required, relevant to the species they care for [82]	Yearly
NSW	Biodiversity Conservation Act 2016 (NSW) Required [10] (No 63 Division 3. 2.17. {f})	Code of Practice for Injured, Sick, and Orphaned Protected Fauna (NSW). New fauna rehabilitators must undertake an introductory training course. Fauna rehabilitators must attend an advanced training course every three years [9] (13.1.1.)	As specified in the licence
NT	Territory Parks and Wildlife Conservation Act 2014 (NT) Permit required [15] (Division 6. 55 {1})	Territory Parks and Wildlife Conservation Act 2014 (NT). Must have previous experience, qualifications, membership to a local wildlife care group, an experienced mentor, facilities, and resources. An application for a permit may be refused if these requirements are not met [15]	Bi-annually
QLD	Nature Conservation Act 1992. (Qld) Rehabilitation permit required [17] (Pt 5. 88 {2})	Nature Conservation Act 1992. (QLD) Individuals must be experienced in wildlife care/rehabilitation or obtain endorsement by wildlife care group under their license. Specialist species require two referee reports from people of professional standing in the relevant wildlife management field [17] (Pt 5. 88 {2})	Nature Conservation Administration Regulation 2017 (Qld) Tri-annually or for the life of a protected animal kept under the permit [80] (Section 21 p. 17 e)
SA	National Parks and Wildlife Act 1972 (SA) Permit required. The Minister may grant to any person a permit to take and hold protected animals or the eggs of protected animals, if satisfied that it is desirable to grant the permit [21] (Section 53)	General Guidelines for the Management of Protected Wildlife in Captivity in South Australia 2010 (SA). Permit will only be issued if the applicant can demonstrate that they have the necessary skills, experience, and resources and resides in SA [23] (Section 4.1.)	Annually
TAS	Wildlife Regulations 1999 (TAS) Yes. A licence is required except for brushtail possum, Bennett’s wallaby, and Tasmanian pademelon [27] (Part 2.5.)	Wildlife (General) Regulations 1999 (TAS). Permit to rehabilitate wildlife only given after vetting process via registration form	Variable. Given with regard to the time an animal is expected to be in care
VIC	Wildlife Act 1975 (Vic) A wildlife shelter permit is required for the purposes of wildlife rehabilitation [31] (Section 28)	Code of Practice for the Welfare of Wildlife during Rehabilitation 2017 (Vic) Wildlife rehabilitators need to demonstrate that they have acquired appropriate training or the required knowledge [33]	Choice of one year or three years
WA	Wildlife Conservation Regulations 1970 (WA) Keeping of fauna in captivity. No permit or licence required. A person may temporarily keep in captivity or confinement fauna that is sick, diseased, or injured or that is abandoned juvenile fauna, for the purpose of caring for it until it recovers or becomes capable of fending for itself [37] (Part 4 28A {1})	Not required	Not applicable

**Table 4:** Standards of care, facility requirements, and identifying of animals in the states and territories of Australia.

State/Territory	Code of Practice Issued	Provision of Facilities	Animal Identification
ACT	No (In progress)	Must provide all equipment and some food costs [82] (para. Equipment)	Rings for identification allowed on birds, otherwise not required
NSW	Yes	The Department of Environment, Climate Change, and Water will not provide recompense for expenses incurred by rehabilitators [11]	Yes. Fauna rehabilitators encouraged to mark animals [73] (section 2.36)
NT	No code, but individual guides available, e.g., about caring for wildlife, caring for macropods, caring for raptors [14]	Wildlife carer’s responsibilities. A wildlife carer’s work is voluntary and costs for food, bedding, cages, equipment, and vets must be met by the carer [14]	Can be required to do so [16]
QLD	Yes [19,91]	All costs met by wildlife carers [19]	No, except if authorised [19]
SA	Yes [23]	All costs associated with the rescue, transport, and rehabilitation of protected wildlife are to be met by the individual carers [23]	No requirement
TAS	Yes [28]	Funding for feed, housing, veterinary care, and emergency situations is the responsibility of the individual carer [28]	Can be instructed to mark the animal [26]
VIC	Yes [33]	All costs met by wildlife carers but grants are available to help with infrastructure and training costs [93]	Must not mark animals without a licence [31]
WA	Yes [38]	No government funding.	No requirement

**Table 5:** Funding and monitoring of wildlife carers and carer networks in Australia.

State/Territory	Financial Support Provided by State/Territory Government	Inspection of Premises and Records	Compliance and Legal Issues
ACT	No	Enforcement powers given [96] (Part 7 Divisions 7.3 and 7.4)	Obtaining Court orders and corporate penalties [96] (Part 7 Division 7.11)
NSW	Some funding given to licensed groups.	At least once every three years [11]	A person who contravenes a condition of a biodiversity conservation license is guilty of an offence [10] (No 63. 2.14 {4})
NT	Funding of AUD$50,000 per year divided between three organisations [97]	Inspection every two years [15]	The role of Conservation Officers is to implement and enforce compliance [15]
QLD	None by State. Some grants available from local councils, e.g., Brisbane [98]	Inspections may be carried out as part of a new permit application assessment, information or complaints received from the public or randomly selected audits on permit holders [18]	The Department of Environment and Science is responsible for the assessment and licensing of individuals and organisations [99]
SA	None.	Wardens can undertake random inspections [22]	Failure to comply, penalties, regulations and codes of practice enforced by wardens of the Department of Environment and Natural Resources [21]
TAS	Management of wildlife carers provided by the State Department of Primary Industries Parks, Water and Environment. No other funding given.	Random checks may occur [28]	An authorised officer may inspect facilities and records [28]
VIC	Wildlife rehabilitator grants of up to AUD$2000 per applicant available. 2018/2019 A total AUD$170,000 has been allocated [93]	Inspections are often conducted to monitor general compliance trends among authorisation holders [100]	Authorised Officers have the power to enter, inspect, or search any property and any buildings or structures other than a dwelling, as well as vehicles or boats, with or without notice [31]
WA	No funding given	No inspection requirement.	Not monitored.

**Table 6:** List of advice given to Australian wildlife carers for pre-release behavior conditions. When not identical, similarities in advice have been placed into a single item.

Element of Advice	NT	NSW	QLD	SA	TAS	VIC	WA
Fauna undergoing rehabilitation must be prevented from coming into contact with domestic pets/animals	✓	✓	✓		✓	✓	✓
Housing must be designed and/or positioned so that fauna cannot see domestic pets or incompatible species	✓	✓	✓		✓		✓
Housing must be positioned so that fauna is not exposed to strong vibrations, noxious smells, or loud noises		✓	✓		✓	✓	✓
Failure to recognise pet species as predators will preclude rehabilitated wildlife from being released into the wild. Animals are not suitable for release unless they display instinctual fear and avoidance towards humans and domestic pets	✓	✓		✓	✓	✓	✓
Hand-reared gregarious species must be exposed to members of the same species or family		✓	✓		✓	✓	✓
During pre-lease, exposure to humans should be greatly reduced	✓	✓			✓	✓	✓
Species that manipulate their physical environment, e.g., dig burrows or build nests, should be given an opportunity to do so		✓	✓	✓	✓		
Enclosures must allow for the display of natural behaviours and sufficient room to avoid ‘stress’ behaviours. They should be large enough for the animal to learn or relearn behaviours, and if occupied by several animals of the same species must be large enough to allow for normal patterns of group behaviour	✓	✓	✓	✓	✓	✓	✓
Prior to release food must be offered in a way that encourages natural feeding behaviour. Good feeding management is essential for maximum development of natural behaviour and survival techniques		✓	✓	✓	✓	✓	✓
Housing should be provided in such a way as to enable training for survival in the wild						✓	

**Table 7:** Published Australian protocols for releasing or not releasing rehabilitated native fauna to the wild.

State/Territory	Release to the Wild Required	Assessment of Suitability for Release Required	Action on Non-Releasable Wildlife	Allowed to Be Kept Permanently in Captivity
ACT	Yes [96] (Section 18 Pt 2 Section 3)	No	Non-releasable animals which are inappropriate for education, foster-parenting, or captive breeding have a right to euthanasia [89]	Yes, under conditions [2]
NSW	A biodiversity conservation licence under Division 3 required [10] (No 63 Part 2.6)	Yes. To be carried out by a wildlife veterinarian or an experienced rehabilitator [11]	Animal must be euthanased or referred to the Department of Environment, Climate Change and Water [11]	Yes, given specified conditions can be met [11]
NT	Must release to wild unless a permit to keep is issued [14]	No	Euthanase [14]	Yes. Permit required [14]
QLD	Only if wildlife is assessed as physically and behaviourally fit to be released [91]	Yes. To be carried out by a wildlife veterinarian or an experienced rehabilitator [91]	Euthanase or refer to authority [19]	Yes. Permit to keep wildlife can be issued under specified purposes or a recreational wildlife licence to keep a prescribed protected animal for recreational purposes (i.e., as a pet) [80]
SA	National Parks and Wildlife Act 1972 (SA) Permit to release required [21]	No. The release of long-term captive animals is rarely justified on conservation or animal welfare grounds [101]	Rescued protected wildlife which cannot be released or retained with a good expectation of quality of life must be euthanased	Permits are available to take protected wildlife from the wild, to keep wildlife as pets and to buy and sell wildlife [21]
TAS	The Secretary must be notified prior to the intended release of any animal referred to on a permit [28]	Details of the process for the release of protected wildlife and the common wombat should be provided to conservation branch prior to release [28]	No direction given	Permits required for partly or wholly protected species Brushtail possums, Bennett’s wallabies and pademelons can be kept without a permit [27]
VIC	Victorian Wildlife Shelter and Foster Carer Authorisation Guide Condition 21. Yes, must release wildlife within 24 h of it being ready for release (condition 21)	All animals to be released must be inspected by a veterinarian or experienced wildlife rehabilitator [33]	Wildlife that cannot be released must be euthanased [100] (condition 22)	No. Note: only legal to keep certain classes of wild pigs as pets [31,32]
WA	Yes. A person who keeps fauna under sub regulation (1) must release it	Self-regulated. Advice on release evaluation given [38]	Must give the animal to appropriate authority	Not allowed [36]

**Table 8:** Standards of health and behaviour required of rehabilitated wildlife in various jurisdictions prior to release in Australia.

	NSW	NT	QLD	SA	TAS	VIC	WA
**Health**							
1. Recovered from injury and/or disease	✓	✓	✓	✓	✓	✓	✓
2. Not known to be sterile/unable to reproduce			✓				
3. Weight and condition are within an appropriate range	✓	✓	✓		✓		✓
4. Appropriate fitness levels	✓	✓		✓	✓	✓	✓
5. Not a biosecurity risk				✓			
6. Pelage, plumage, scales or skin is adequate for survival	✓	✓					✓
7. Acclimated to prevailing climatic conditions	✓		✓		✓	✓	
8. Must be independent of its natural mother					✓		
9. Salt tolerant, for marine species	✓						
10. Of sufficiently mature age for independent survival							✓
11. Weaned off all unnatural feedstuffs					✓		
12. Must be released before sexual maturity					✓		
**Behaviour**							
13. Can recognise, catch and consume appropriate, naturally available food	✓	✓	✓	✓	✓	✓	✓
14. Can recognise and successfully avoid predators and domestic carnivores	✓		✓		✓	✓	✓
15. Not attracted to humans or sights, sounds, or smells that are specific to captivity	✓		✓				
16. Show a fight or flight response similar to that shown by wild conspecifics		✓				✓	✓
17. Can navigate effectively through its natural environment	✓		✓		✓	✓	✓
18. Can recognise and interact normally with other members of the same species	✓	✓	✓		✓	✓	
19. Mark its territory, if applicable						✓	✓
20. Find or construct shelter						✓	✓

**Table 9:** Pre-release and post-release behaviour methodology and monitoring of native wildlife in care, as proposed in the states and territories of Australia.

State/Territory	Pre-Release Behaviour	Programmed Behaviour Modification Before Release	Post-Release Monitoring
ACT	Code of ethics. Releasable native fauna should be maintained in a wild condition and released as soon as appropriate [89]	None specified	No
NSW	Comprehensive advice given [9] (8.2.1.5. &10.1.1.4.)	None specified	Encouraged [107] (12.3.1.1&4)
NT	Advice on housing and avoiding interaction with pets and humans [14]	None specified	None
QLD	Comprehensive advice given [91]	None suggested	Few studies undertaken
SA	Advice given not to humanise or allow the animal to be imprinted [23]	None specified	None
TAS	Comprehensive advice given [28]	None specified	None
VIC	Comprehensive advice given [104]	Housing should be provided in such a way as to enable training for survival in the wild [104]	None specified
WA	Advice on pre-release conditioning given [38] (Chapter 1. (7) Stage 3)	None specified	Suggested [38] (Chapter 1 {9})
